# Supplementary material for: An Efficient and Universal Protoplast Isolation Protocol Suitable for Transient Gene Expression Analysis and Single-Cell RNA Sequencing
Source: Int J Mol Sci. 2022 Mar 22;23(7):3419. doi: 10.3390/ijms23073419 (PMC8998730; doi:10.3390/ijms23073419)
Supplement: Supplementary file 1 [file ijms-23-03419-s001.zip › Wang-Supplemental Figures and Tables 2022-03-20.pdf]

An efficient and universal protoplast isolation protocol suitable for transient gene expression analysis and single-cell RNA sequencing

Juan-Juan Wang, Yang Wang, Tian-Feng Lü, Xia Yang, Jing Liu, Yang Dong and Yin-Zheng Wang

Supplemental Figure S1-S7 and Supplemental Table S1 and S2

Supplemental Figure S1. Statistical analysis of protoplast quality with and without pretreatment procedure

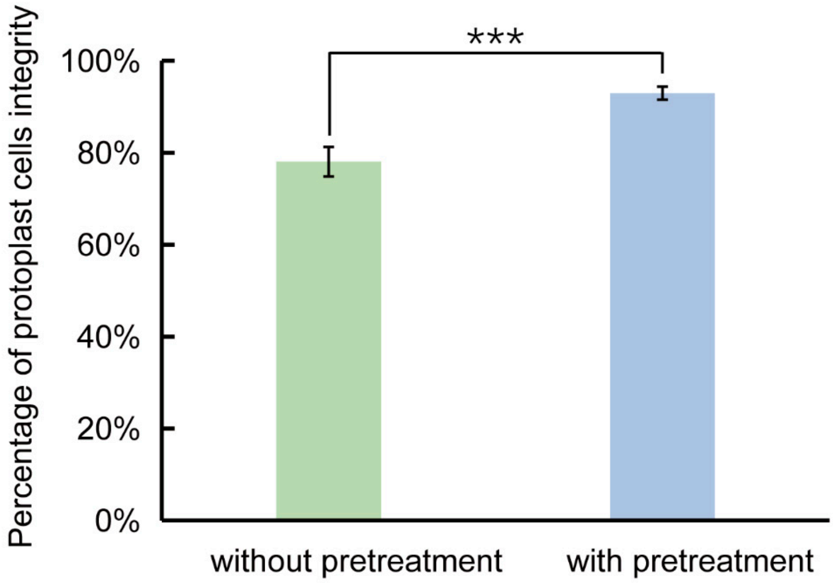

The protoplast quality was evaluated as the percentage of round and green single cells relative to the total protoplasts in the observation field. Please be noted that the protoplasts were immediately subjected to quality check after isolation. The values (mean ± standard deviation) are the average of three replicates with each replicate containing 10-20 observations. The statistical significance test was performed with Student's *t* test (***, *p* value <0.001).

Supplemental Figure S2. Phylogenetic analysis of genes involved in the epigenetic modification machinery

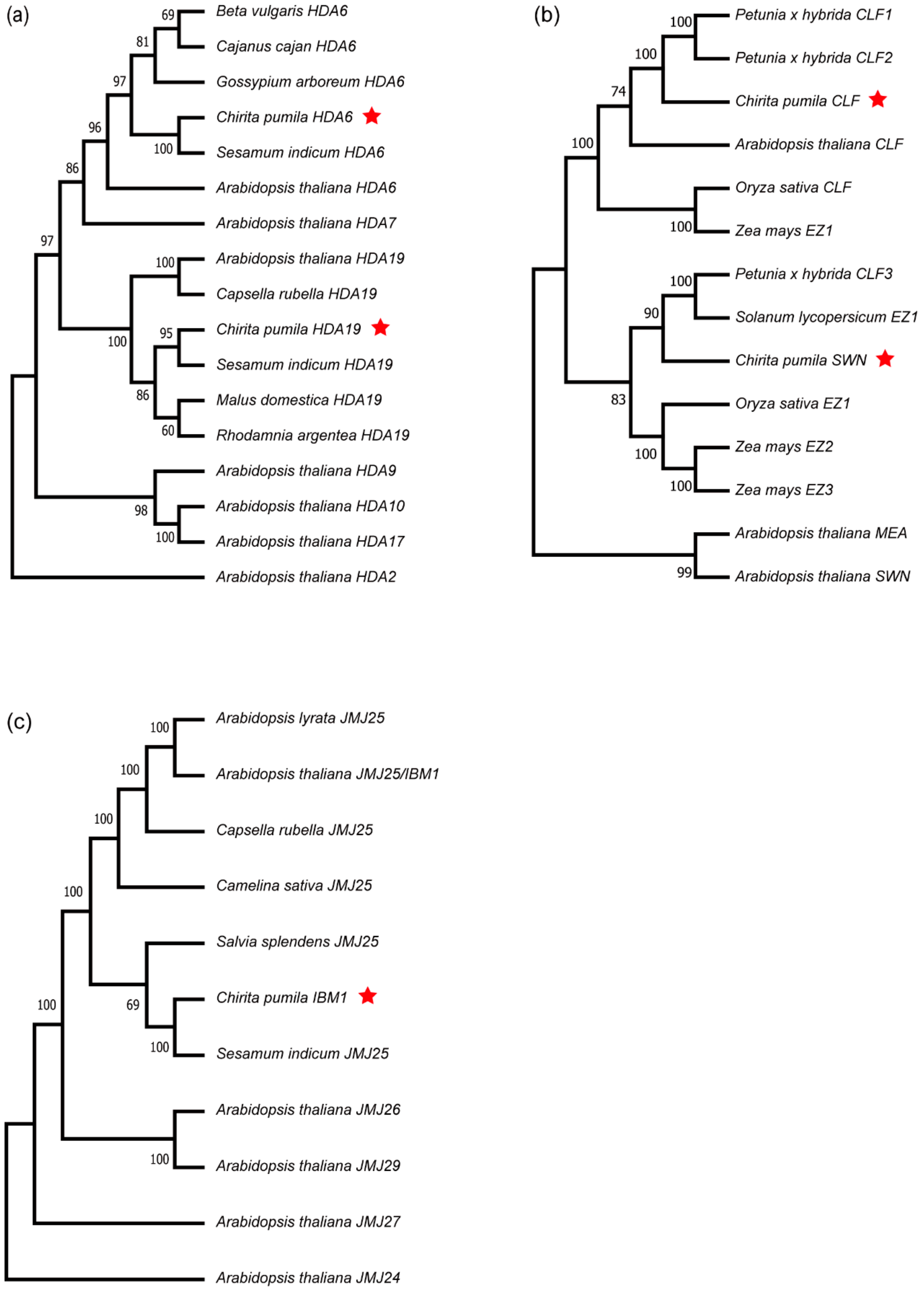

Neighbor-Joining phylogenetic trees of *CpHDA6* and *CpHDA19* (a), *CpCLF* and *CpSWN* (b) and *CpIBM1* (c). The red stars indicate the orthologous sequence of *Arabidopsis* counterpart in *C. pumila*.

Supplemental Figure S3. Expression analysis of genes involved in the epigenetic modification machinery between the digested protoplasts and unprocessed leaf samples in *C. pumila*

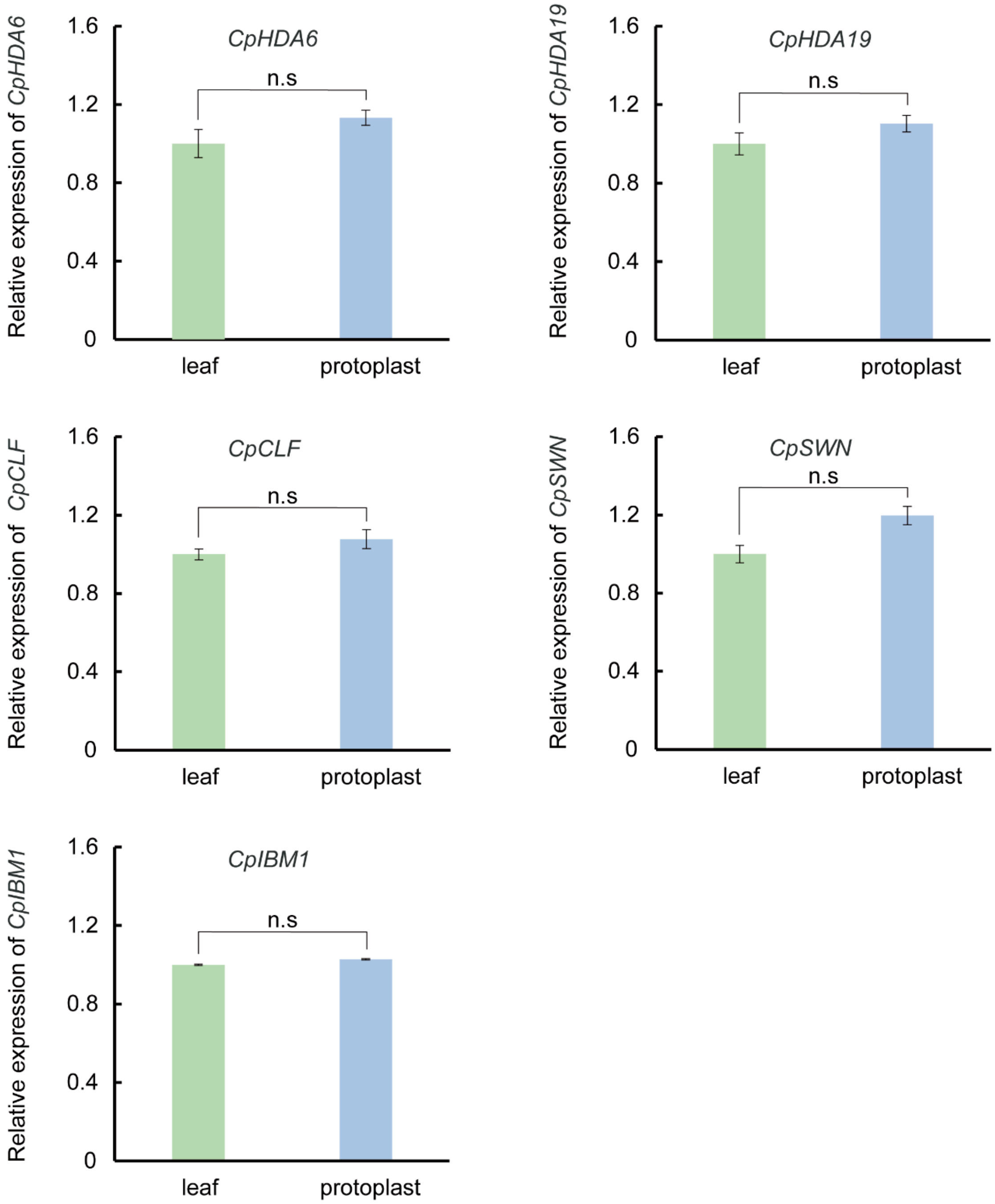

The values shown are the average of three biological replicates. The expression level of each gene in the protoplast were normalized to the relative expression level in the unprocessed leaf samples. The statistical significance test was performed with Student's *t* test (n.s. indicate non-significant, p>0.05).

Supplemental Figure S4. Viability assay of *C. pumila* mesophyll protoplasts by time-lapse FDA staining.

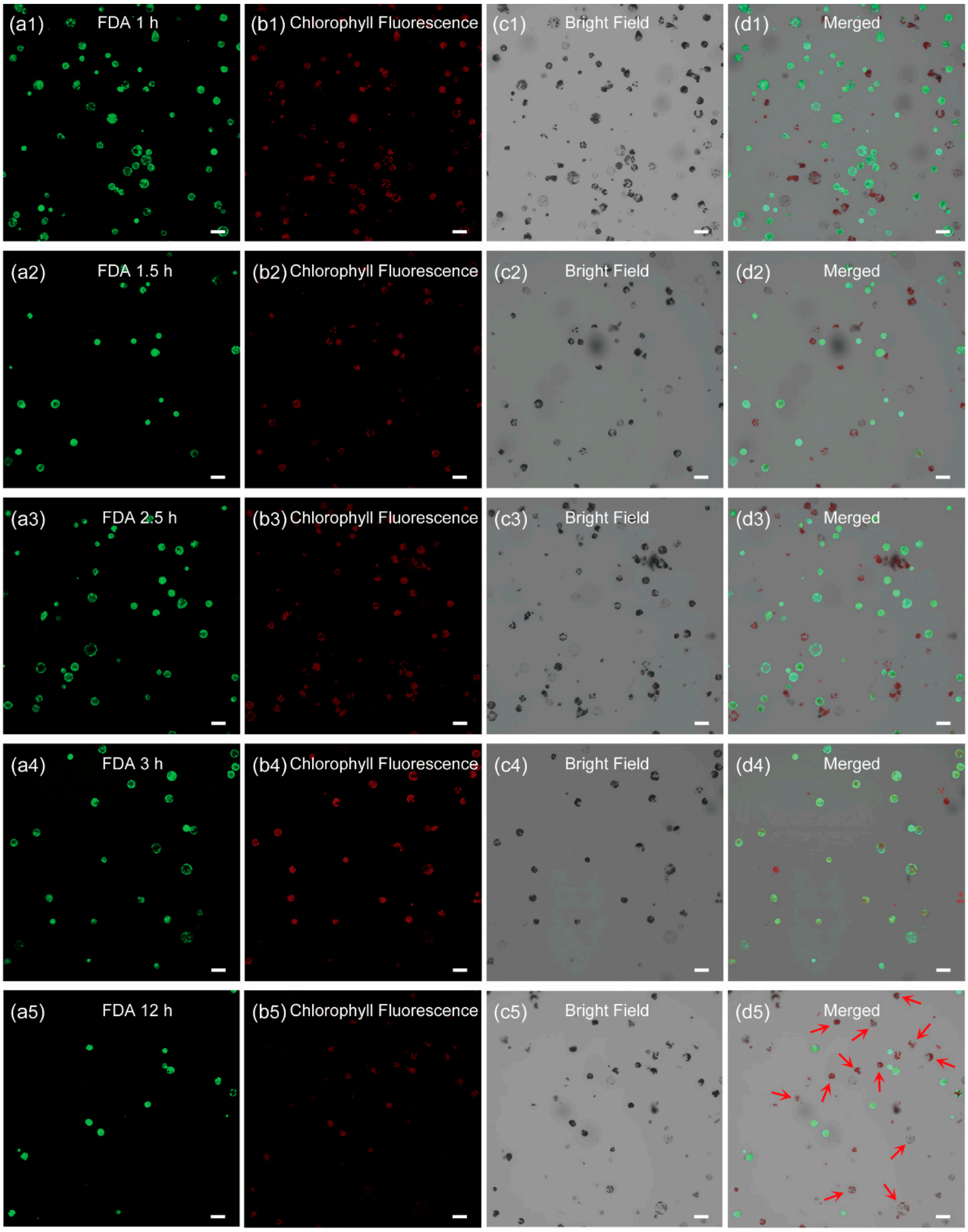

Protoplasts stained with 0.1mg/L FDA were imaged in a time-lapse series (1.0 h, 1.5 h, 2.5 h, 3 h and 12 h). The pictures were taken under GFP channel (a1-a5), chlorophyll auto-fluorescence channel (b1-b5), bright field (c1-c5) and merged channel (d1-d5), respectively. Please be noted that the cell viability was significantly reduced in 12h as indicated the disintegrated cell membrane system (red arrows) possibly due to the existence of acetone in the FDA buffer. Scale bars, 50 μm.

Supplemental Figure S5. Viability assay of *C. pumila* mesophyll protoplasts by time-lapse rhodamine 123 staining.

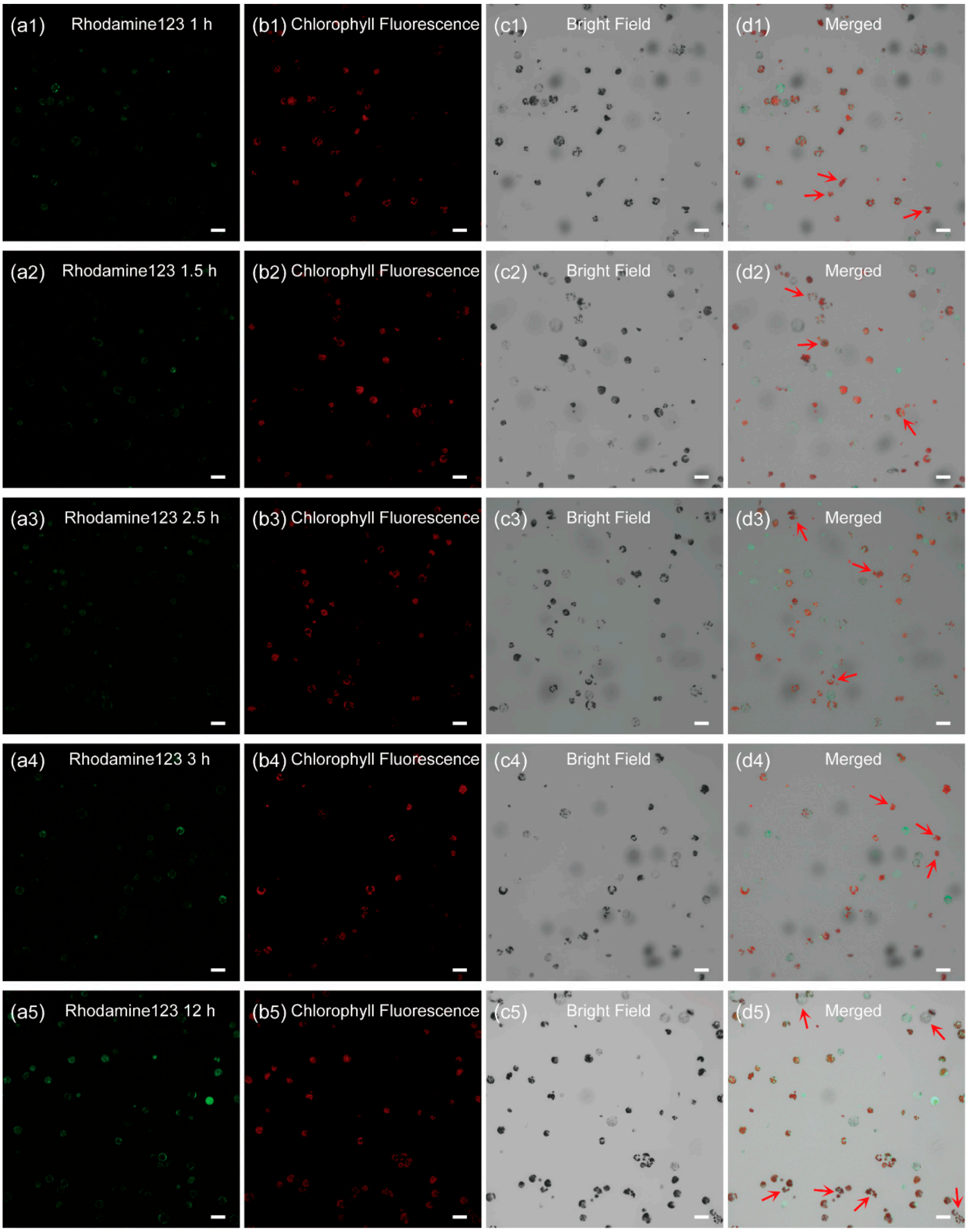

Protoplasts stained with rhodamine 123 (final concentration of 1 μM) were imaged in a time-lapse series (1.0 h, 1.5 h, 2.5 h, 3 h and 12 h). The pictures were taken under GFP channel (a1-a5), chlorophyll auto-fluorescence channel (b1-b5), bright field (c1-c5) and merged channel (d1-d5), respectively. The red arrows indicate cells without GFP signals, which are associated with disintegrated cell membrane system. Scale bars, 50 μm.

Supplemental Figure S6. Statistical analysis of protoplast viability with FDA and rhodamine 123 staining, respectively.

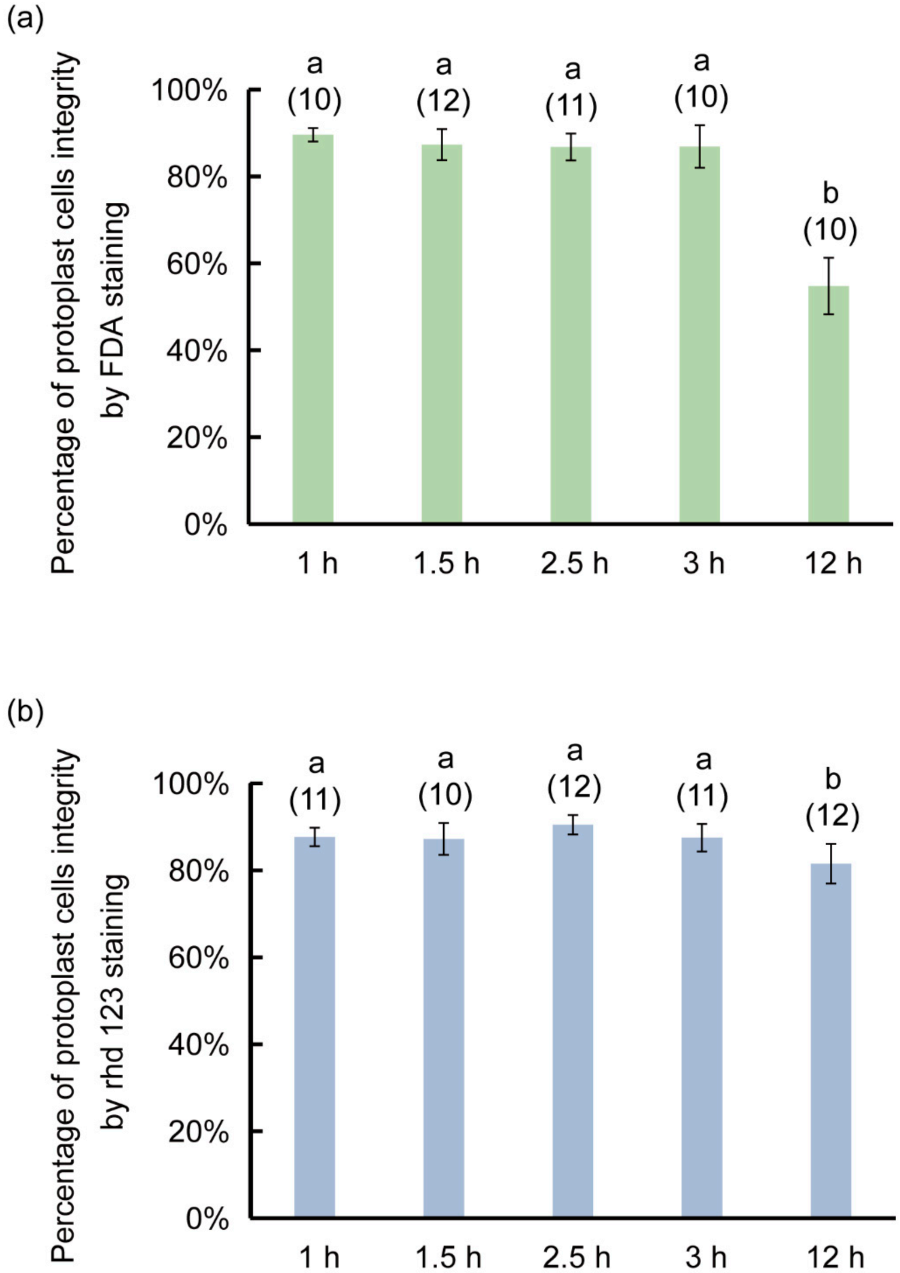

The protoplast viability was calculated as the percentage of round cells with GFP signals for FDA staining and clear GFP signals for rhd123 staining, respectively, in a given observation field. The values (mean ± standard deviation) are the average of three replicates with each replicate containing 10-20 observations. The statistical significance test was performed with Student's *t* test (a, non-significant, $p>0.05$; b, p value <0.001 in (a) and p value <0.05 in (b)).

Supplemental Figure S7. Enlarged images of Figure 6

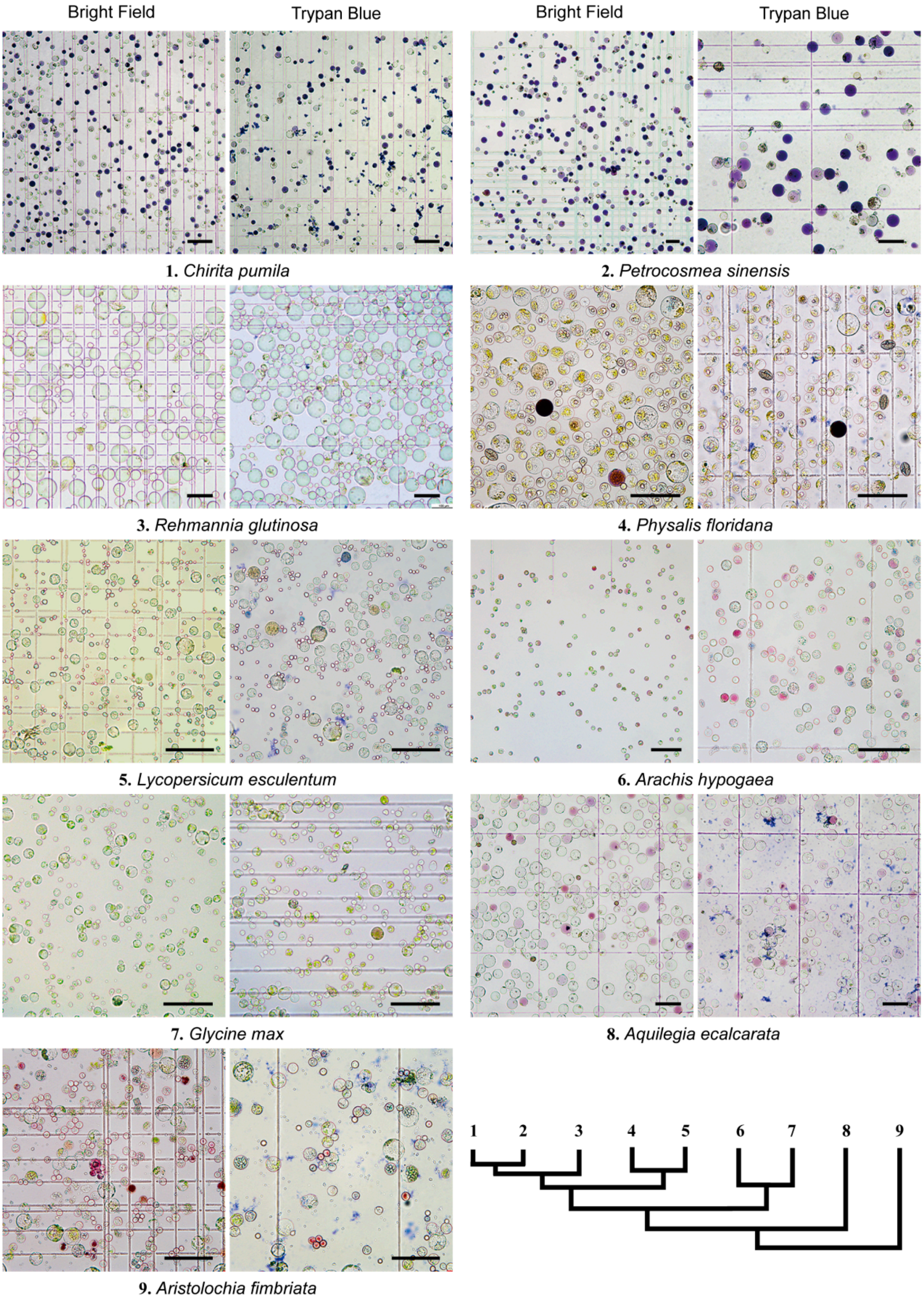

The phylogenetic relationship of the representative species was based on Angiosperm Phylogeny Group (APG) IV. Please be noted that some of cells are in dark purple or magenta color due to anthocyanin biosynthesis in the samples. Scale bars, 100 µm.

Supplemental Table S1. List of oligos used in this study

Primer name	Sequences (5’-3’)	Primer annotation
35S mini (-46bp)	agctctcgag**AAGCTT**CTCTATATAA -GGAAGTTCATTTCATTTGGAGAGAACA -CGGGGGACT**GGATCC**atggtgagca	Plasmid construction
4×ABRE	agctctcgag**AAGCTT**TGCGTACGTGTT -GTTTGCGTACGTGTTGTTTGCGTACGTGTTG -TTTGCGTACGTGTTGTTagcttctctatataa	Plasmid construction
4×mABRE	agctctcgag**AAGCTT**TGCGAATCAATT GTTTGCGAATCAATTGTTTGCGAATCAATTGT -TTGCGAATCAATTGTTagcttctctatataa	Plasmid construction
AREB1-F	tccccttgctccgt**GGATCC**TTC -ATGAATTTCGGGAACG	Plasmid construction
AREB1-R	aatgtttgaacgat**CTGCAG**CCAGAAT -TGGAGGTACTAAGATAAG	Plasmid construction
p35S-GUS-F	tccccttgctccgt**GGATCC**GGACTCT -TGACCATGGTAGATCTGAG	Plasmid construction
p35S-GUS-R	aatgtttgaacgat**CTGCAG**GGTCACCTG -TAATTCACACG	Plasmid construction
GFP-RT-F	GCTGCTGGGATTACACATGGCATGG	Gene expression analysis
GFP-RT-R	GATAATCATCGCAAGACCGGCAAC	Gene expression analysis
GUS-RT-F	CGCTGGACTGGCATGAACTTCGGTG	Gene expression analysis
GUS-RT-R	GGATCCCCAATTCCCGAGGCTG	Gene expression analysis
CpACT-RT-F	AGTTATCACCATTGCCGCCGAGAGG	Gene expression analysis
CpACT-RT-R	GCAATGCCAGGGAACATAGTCGACC	Gene expression analysis
CpIBM1-RT-F	GGATGACGAGGCTGTGGTAGATG	Gene expression analysis
CpIBM1-RT-R	TCATGTGCACCATTGGTTTTATCACT	Gene expression analysis
CpHDA6-RT-F	GGAACAGGGCACATCAAAGACA	Gene expression analysis
CpHDA6-RT-R	GAATCTGCTCCACATTGAAGAACAAC	Gene expression analysis
CpHDA19-RT-F	GGCAATTCACTTCCATCTGGAAC	Gene expression analysis
CpHDA19-RT-R	CAGTAATCCGTAGTGAGCAAGAAGAGC	Gene expression

		analysis
CpCLF-RT-F	TTATTGGCTCTGATCGAAGTGTGGA	Gene expression analysis
CpCLF-RT-R	TGGACGAACGGCATTCTTGACT	Gene expression analysis
CpSWN-RT-F	AACCTATGTTCTTGACGCTTACCG	Gene expression analysis
CpSWN-RT-R	TTCTCCAGCTTCAATACGCTCATT	Gene expression analysis

Lowercase letters are adaptor sequences for in-fusion HD cloning and restriction enzyme sites are shown with bold capital letters (*Hind*III: **AAGCTT;** *Bam*HI: **GGATCC**; *Pst*I: **CTGCAG**).

Supplemental Table S2. Accession numbers of genes used for the phylogenetic analysis

Species	Gene	ID
Arabidopsis thaliana	*HDA2*	832673
Arabidopsis thaliana	*HDA6*	836431
Arabidopsis thaliana	*HDA7*	833525
Arabidopsis thaliana	*HDA10*	823592
Arabidopsis thaliana	*HDA17*	823574
Arabidopsis thaliana	*HDA19*	829969
Arabidopsis thaliana	*HDA9*	823594
Beta vulgaris	*HDA6*	104894337
Cajanus cajan	*HDA6*	109798024
Capsella rubella	*HDA19*	17879361
Gossypium arboreum	*HDA6*	108483673
Malus domestica	*HDA19*	103428059
Rhodamnia argentea	*HDA19*	115756819
Sesamum indicum	*HDA19*	105165913
Sesamum indicum	*HDA6*	105179380
Arabidopsis lytata	*JMJ25*	9320727
Arabidopsis thaliana	*JMJ24*	837427
Arabidopsis thaliana	*JMJ25/IBM1*	819952
Arabidopsis thaliana	*JMJ26*	837747
Arabidopsis thaliana	*JMJ27*	827933
Arabidopsis thaliana	*JMJ29*	842529

Camelina sativa	*JMJ25*	104793410
Capsella rubella	*JMJ25*	17890836
Salvia splendens	*JMJ25*	121744023
Sesamum indicum	*JMJ25*	105178012
Arabidopsis thaliana	*CLF*	816870
Arabidopsis thaliana	*MEA*	839422
Arabidopsis thaliana	*SWN*	828165
Oryza sativa	*CLF*	4340748
Oryza sativa	*EZ1*	4332612
Petunia x hybrida	*CLF1*	AB098523.1
Petunia x hybrida	*CLF2*	AB098524.1
Petunia x hybrida	*CLF3*	AB098525.1
Solanum lycopersicum	*EZ1*	100134891
Zea mays	*EZ1*	541954
Zea mays	*EZ2*	542659
