# Supplementary material for: An Efficient and Universal Protoplast Isolation Protocol Suitable for Transient Gene Expression Analysis and Single-Cell RNA Sequencing
Source: Int J Mol Sci. 2022 Mar 22;23(7):3419. doi: 10.3390/ijms23073419 (PMC8998730; doi:10.3390/ijms23073419)
Supplement: Supplementary file 1 [file ijms-23-03419-s001.zip › Wang-Supplemental Method 2022-03-20.pdf]

An efficient and universal protoplast isolation protocol suitable for transient gene expression analysis and single-cell RNA sequencing

Juan-Juan Wang, Yang Wang, Tian-Feng Lü, Xia Yang, Jing Liu, Yang Dong and Yin-Zheng Wang

Supplemental Method

Reagent setup and Step-by-step method details

Experimental considerations

1. Before starting this protocol, please ensure that instruments and equipment required for protoplast extraction at 25 ˚C are available as these steps are crucial for the successful protoplast isolation.
2. It should be noted that this protocol is set up and optimized for *Chirita pumila*, but may also be applicable to other non-model organisms.

1. Reagent setup

a. Prepare 2 M KCl (10 mL)

Dissolve 1.491 g KCl with 8 mL double distilled water (ddH_2O), and then fill up to 10 mL with ddH_2O. Autoclave at 121 ˚C for 20 mins. Store at room temperature (25 ˚C) up to 4 weeks.

b. Prepare 1 M $CaCl_2$ (20 mL)

Dissolve 2.22 g $CaCl_2$ with 15 mL ddH_2O, and then fill up to 20 mL with ddH_2O. Sterilize using 0.22 μm sterile membrane filters in a laminar hood. Store at room temperature (25 ˚C) up to 4 weeks.

Note: Calcium chloride can be sterilized using 0.22 μm filters or autoclaving at 121 ˚C for 20 mins.

c. Prepare 2 M $MgCl_2$ (20 mL)

Dissolve 8.132 g $MgCl_2{\cdot}6H_2O$ in 15 mL ddH_2O, and then fill up to 20 mL with ddH_2O. Sterilize with 0.22 μm membrane filter. Store at room temperature (25 ˚C) up to 4 weeks.

d. Prepare 0.5 M NaCl (100 mL)

Dissolve 2.922 g NaCl in 80 mL ddH_2O, and then fill up to 100 mL with ddH_2O. Autoclave at 121 ˚C for 20 mins. Store at room temperature (25 ˚C) up to 4 weeks.

e. Prepare 0.2 M 2-(N-Morpholino) ethanesulfonic acid (MES) (100 mL)

Dissolve 4.264 g with 80 mL ddH_2O, adjust pH to 5.7 with KOH and fill up to 100 mL with ddH_2O. Sterilize using 0.22 μm filters in a laminar hood. Aliquot (10 mL for each) and store at 4 ˚C for up to 2 weeks.

f. Prepare 0.8 M mannitol (100 mL)

Dissolve 14.576 g mannitol with 80 mL ddH_2O, and then fill up to 100 mL with ddH_2O. Sterilize using 0.22 μm filters in a laminar hood. Store at 4 ˚C for up to 4 weeks.

Note: Mannitol is very prone to be contaminated by bacterial and mold, please conduct this step in a laminar hood. Aliquot (10 mL for each) and store at 4 ˚C for up to one month.

g. Prepare 5% Bovine serum albumin (BSA)

Dissolve 0.5 g BSA in 10 mL ddH_2O. Sterilize using 0.22 μm filters in a laminar hood. Aliquot (1 mL for each) and store at -20 ˚C up to 4 weeks.

Note: Avoid repeated freezing and thawing. The BSA buffer can be stored at 4 ˚C for up to one week.

- **Protoplast isolation related buffers**

✧ **Prepare the Pretreatment Buffer (10 mL)**

Mix 100 μL 2 M KCl, 100 μL 1 M $CaCl_2$,1 mL 0.2 M MES, 7.5 mL 0.8 M mannitol and 200 μL 5 % BSA in 10 mL sterilized water.

Reagent	Final concentration	Amount
2 M KCl	20 mM	100 μL
0.2 M MES	20 mM	1 mL
0.8 M mannitol	0.6 M	7.5 mL
1 M $CaCl_2$	10 mM	100 μL
5% BSA	0.1 %	200 μL
ddH_2O		up to 10 mL

Note: Prepare fresh pretreatment buffer before each experiment.

✧ **Prepare Enzyme Buffer I (100 mL)**

Dissolve 1.0 g cellulase R10, 0.5 g macerozyme R10, 0.5 g pectinase in 8 mL ddH_2O, add 1 mL 2 M KCl, 10 mL 0.2 M MES and 75 mL 0.8 M mannitol to the mixture in the order specified. Heat up the mixture to 55 ˚C for 10 mins in a water bath to deactivate the proteases and cool down the mixture to room temperature. Add 1 mL 1 M $CaCl_2$ and 2 mL 5 % BSA into the buffer and mix gently. Add ddH_2O up to 100

mL.

Reagent	Final concentration	Amount
cellulase R10	1 %	1 g
macerozyme R10	0.5 %	0.5 g
pectinase	0.5 %	0.5 g
ddH_2O		8 mL
2 M KCl	20 mM	1 mL
0.2 M MES	20 mM	10 mL
0.8 M mannitol	0.6 M	75 mL
55 ˚C for 10 mins in water bath then cool down to room temperature		
1 M $CaCl_2$	10 mM	1 mL
5% BSA	0.1 %	2 mL
ddH_2O		up to 100 mL

Note: Sterilize the buffer using 0.22 µm filters in a laminar hood. Aliquot (10 mL for each) and store at -20 ˚C. Thaw on the ice and mix thoroughly before use.

✧ **Prepare Enzyme Buffer Π (100 mL)**

Dissolve 1.2 g cellulase R10, 0.4 g macerozyme R10 with 8 mL ddH_2O, add 1 mL 2 M KCl, 10 mL 0.2 M MES, 75 mL 0.8 M mannitol to the mixture. Heat up the mixture to 55 ˚C for 10 mins in a water bath to deactivate the proteases and cool down the mixture to room temperature. Add 1 mL 1 M $CaCl_2$ and 2 mL 5 % BSA into the buffer and mix gently. Add ddH_2O up to 100 mL.

Reagent	Final concentration	Amount
cellulase R10	1.2 %	1.2 g
macerozyme R10	0.4 %	0.4 g
ddH_2O		8 mL
2 M KCl	20 mM	1 mL
0.2 M MES	20 mM	10 mL
0.8 M mannitol	0.6 M	75 mL
55 ˚C for 10 mins in water bath then cool down to room temperature		
1 M $CaCl_2$	10 mM	1 mL
5% BSA	0.1 %	2 mL
ddH_2O		up to 100 mL

Note: Sterilize the buffer using 0.22 µm filters in a laminar hood. Aliquot (10 mL for each) and store at -20 ˚C. Thaw on the ice and mix thoroughly before use.

✧ **Prepare W5 Buffer (50 mL)**

Mix 0.125 mL 2 M KCl, 6.25 mL 1 M $CaCl_2$, 15.4 mL 0.5 M NaCl, 0.5 mL 0.2 M MES with ddH_2O and then fill up to 50 mL with ddH_2O. Store at room temperature (25 °C) up to 2 weeks.

Reagent	Final concentration	Amount
2 M KCl	5 mM	0.125 mL
1 M $CaCl_2$	125 mM	6.25 mL
0.5 M NaCl	154 mM	15.4 mL
0.2 M MES	2 mM	0.5 mL
ddH_2O		up to 50 mL

Note: W5 solution should be freshly prepared right before the experiment.

- **Protoplast transformation related buffers**

✧ **Prepare MMG Buffer (5 mL)**

Mix 3.75 mL 0.8 M mannitol, 37.5 μL 2 M $MgCl_2$, 0.1 mL 0.2 M MES with ddH_2O up to 5mL. Prepare and mix well this solution before use.

Reagent	Final concentration	Amount
0.8 M mannitol	0.6 M	3.75 mL
2 M $MgCl_2$	15 mM	37.5 μL
0.2 M MES	4 mM	0.1 mL
ddH_2O		up to 5 mL

Note: MMG buffer should be freshly prepared right before the experiment.

✧ **Prepare 40% PEG solution (1 mL)**

Dissolve 0.4 g PEG4000 with 250 μL 0.8 M mannitol and 200 μL 1 M $CaCl_2$ and add ddH_2O up to 1000 μL. Mix thoroughly by vortexing.

Reagent	Final concentration	Amount
PEG4000	40% (m/v)	0.4 g
0.8 M mannitol	0.2 M	250 μL
1 M $CaCl_2$	0.2 M	200 μL
ddH_2O		up to 1000 μL

Note: Prepare this solution at least 30 mins before transformation. Be sure that PEG4000 is completely dissolved without any bubbles.

2. Step-by-step method details

2.1. Protoplast isolation

TIMING: ~4.5-5 hrs

1. Add 5 mL pretreatment buffer into a 60 mm×15 mm cell culture petri dish.

2. Cut 8-10 young and healthy samples (flowers, tuberous roots, or gonophores or other young tissues) from the plant using a scissor, put the samples in the pretreatment buffer.

Critical: The developmental stage of the sample is crucial for a successful protoplast isolation experiment.

3. Carefully fold the sample (for leaves and flower lips) and slice the sample into ～0.5-1 mm strips using sharp surgical blade in the pretreatment buffer.

Critical: Always slice the sample using a sharp surgical blade from one side to the other. Cutting the sample using scissors will crush the cells at the cutting edge and in turn generate excessive cell rupture. Too much materials also often leads to insufficient enzymatic digestion. To our experience, 10 leaf blade (1 cm^2 each) reach the maximum for 5 mL digestion buffer.

4. Gently submerge the samples into the pretreatment buffer using a tip, penetrate the sample under vacuum for 10 mins.

Critical: This step is performed under vacuum ~100 mbar. Release the vacuum slowly to avoid any damage to plant cells.

5. Discard the pretreatment buffer by pipetting gently.

6. Add 5 mL Enzyme buffer I into the petri dish and gently submerge the sample using a tip.

Critical: Make sure all the samples are submerged by the enzyme buffer, otherwise this will result in poor protoplast yield.

7. Fix the petri dish on a shaker at a speed of 40 rpm and 26 ˚C for 3 hrs.

Critical: To our experience, an excess of the protoplasts would be recovered from the tissues after 3 hrs digestion. Examine the amount of protoplast under the microscope after 3-hour digestion. Proceed to the second-round digestion if the samples were partially digested or only a small number of protoplasts were recovered.

8. Collect the protoplast and partially sample by centrifuging at 25 ˚C, 200 rcf (g) for 3 mins, discard the supernatant completely by gentle pipetting and resuspend the sample with 5 mL Enzyme buffer II.

Critical: The protoplasts are very fragile, please centrifuge the sample with the speed lower than 250 rcf (g) and resuspend the protoplast very gently using blunt tips.

9. Fix the petri dish on a shaker at a speed of 40 rpm and 26 ˚C for 90 mins.

Note: To our experience, a large number of protoplasts will be released after 1-hour digestion.

10. Add 5 mL W5 buffer to the protoplast mixture to stop the reaction.

Critical: The W5 buffer should be added through the edge of the petri dish and gently mix the sample by soft shaking. Avoid any violent movement of the solutions.

11. Wash the 40 μm nylon cell strainer with 5 mL W5 buffer twice, put the nylon cell strainer on a 50 mL falcon tube.

12. Carefully transfer the protoplast mixture onto the cell strainer using blunt tips to filter the protoplast.

Critical: Please use blunt tips as the protoplasts are very fragile.

13. Wash the nylon cell strainer with 2 mL W5 Buffer.

Note: Rinse the digested tissues on the nylon cell strainer with W5 buffer can yield more protoplasts, but more cell debris will be recovered as well.

14. Wash a new 40 μm nylon cell strainer with 5 mL W5 buffer twice, put the nylon cell strainer on a new 50 mL falcon tube.

15. Carefully transfer the protoplasts from step 13 onto the cell strainer using blunt tips to filter the protoplast again.

Critical: Please use blunt tips as the protoplast are very fragile.

16. Collect the protoplast by centrifuging at 25 ˚C, 200 rcf (g) for 3 mins, remove the supernatant completely by gentle pipetting without disturbing the pellet.

Critical: Please centrifuge the protoplast with the speed lower than 250 rcf (g).

17. Resuspend the protoplast with 2 mL W5 buffer by very soft pipetting with a blunt tip.

Critical: Please conduct this step in extremely gentle action and avoid any violent movement of the protoplast.

18. Transfer the protoplast into a 2.0 mL Eppendorf tube.

Critical: Please use blunt tips as the protoplast are very fragile.

19. Place the protoplast on ice for 20 mins to sediment the cells.

Note: To our experience, most of the protoplasts will be sedimented to the bottom of the tube while the cell debris will remain in the supernatant.

20. Remove the supernatant gently and resuspend the protoplasts with 200 μL W5 buffer.

Note: To our experience, resuspension of the protoplast to a final volume of 200 μL will reach a concentration around 1×10^9 cells/mL. Dilute the protoplast to an appropriate concentration according to the downstream experiment requirements.

Critical: Please conduct this step on the ice and in extremely gentle action and avoid any violent movement of the protoplast.

21. Place the protoplast on ice and proceed to the experiment.

2.2. Protoplast quality check (Trypan blue/rhodamine 123 staining/fluorescein diacetate staining, optional)

TIMING: ~10 mins

22. Transfer 20 μL protoplasts to a PCR tube, add 2 μL trypan blue stock solution (0.4%), or 0.2 μL rhodamine 123 solution (100 μM) to a final concentration of 1 μM or 0.2 μL fluorescein diacetate (FDA, 10 mg/mL) stock solution to a final concentration of 0.1 mg/L, mix very gently.

Critical: Please conduct this step gently and avoid any violent movement of the protoplast.

23. Load 10 μL protoplast onto a haemocytometer and examine the cell activity under Zeiss LSM-510 microscope.

Note: The active cells are indicated by round cells without any blue staining and green-fluorescence labeled while using trypan blue staining and fluorescein diacetate (FDA) staining, respectively.

24. Count the number of blue staining protoplasts and the number of total protoplasts.

Note: *% viable protoplasts=[1.00-(Number of blue protoplasts/Number of total protoplasts)]×100, or =(fluorescent protoplast number in view/protoplast total number in view) ×100%

2.3. Protoplast transformation

TIMING: ~45 mins

25. Collect the protoplasts from step 21 by centrifuging at 4 ˚C, 200 rcf (g) for 3 mins, remove the supernatant completely by gentle pipetting without disturbing the pellet.

Critical: Please be aware the temperature for centrifuge is 4 ˚C and make sure that the speed is lower than 250 rcf (g).

26. Resuspend the protoplasts with MMG buffer to the concentration of 1×10^7 cells/mL on the ice.

Critical: Please conduct this step in an extremely gentle action and avoid any violent movement of the protoplast.

27. Mix 10 μL plasmids (1-2 μg/μL) in an appropriate ratio thoroughly in a 2.0 mL Eppendorf tube.

Note: For transient expression assays, we recommend the plasmid ratio of regulatory effectors, reporter and a control reporter in 5:4:1.

Critical: Always prepare the plasmid by Midi prep (Qiagen, 12143) as the plasmid quality is critical for a successful transient expression experiment.

28. Add 100 μL protoplasts prepared from step 26, mix gently with a blunt tip.

Critical: Please conduct this step in extremely gentle action and avoid any violent movement of the protoplasts.

29. Add 110 μL PEG solution to the DNA-protoplasts, mix the mixture by slowly inverting the tubes several times.

Note: The 40% PEG solution should be prepared freshly right before the experiment, ideally between step 19 and 20.

Critical: Please conduct this step in a slow and soft action and avoid any violent movement of the protoplasts.

30. Heat shock the DNA-protoplasts-PEG mixture at 42 ˚C in a water bath for 3 mins.

Note: To our experience, heat shock in the water bath generates better results than in the dry bath.

31. Immediately transfer the tubes in the ice bath and incubate for 10 mins.

32. Add 440 μL W5 buffer to the transformation mixture gently, mix the sample by slowly inverting the tube.

33. Collect the protoplasts from step 32 by centrifuging at 25 ˚C, 200 rcf (g) for 60 seconds (secs), remove the supernatant completely by careful pipetting without disturbing the pellet.

Critical: Please be aware the temperature for centrifuge is 25 ˚C and make sure that the speed is lower than 250 rcf (g).

34. Resuspend the protoplasts with 500 μL W5 buffer by very soft pipetting with a blunt tip.

Critical: Please conduct this step in extremely gentle action and avoid any violent movement of the protoplasts.

35. Collect the protoplasts by centrifuging at 25 ˚C, 200 rcf (g) for 60 secs, remove the supernatant completely by careful pipetting without disturbing the pellet.

Critical: Please make sure that the speed is lower than 250 rcf (g).

36. Resuspend the protoplasts with 1 mL W5 buffer by very soft pipetting with a blunt tip.

Critical: Please conduct this step in extremely gentle action and avoid any violent movement of the protoplasts.

37. Coat the wells in 24-well tissue culture plates with 5% BSA.

Note: Coating 5% BSA in the well is necessary to prevent the protoplasts sticking on the surface.

38. Transfer the cell suspension into the tissue culture plate and culture the protoplasts at 26 ˚C for 16-20 hrs under a light intensity of ～11 µmoL/m^2/s.

2.4 Troubleshooting

Problem 1: Low protoplast yield

- The developmental stages of the plant sample is key to an efficient protoplast release process. Try to choose the samples that are ongoing active growth.
- Slice the samples as finely as you can.
- Make sure the plant materials were fully penetrated by the pre-treatment buffer, and extend the vacuum time to 15 mins in step 4 if necessary.
- Apply vacuum infiltration to step 6 for 10 mins.
- Extend the phase I digestion to 4 hrs if necessary.

Problem 2: Poor cell viability rate

- Check all the buffers and make sure the key buffers are sterile and made fresh.
- Try to finish the protoplast extraction process in 6 hrs.
- If you can acquire enough protoplasts after the first round of digestion, then directly proceed to the following steps without the treatment of enzyme buffer II.
- Try to quantify the cell viability rate immediately after trypan blue staining. Longer staining time will decrease the viability rate.

Problem 3: A substantial amount of cell debris was found in the protoplasts

- Always slice the sample with the sharp surgical blade and avoid any kind of crushing of the samples.
- Always use blunt tips and resuspend the cells with very gently as indicated in the protocol.

- Make sure the centrifuge speed is below 250 rcf (g).
- Sediment the protoplasts in step 19 twice if necessary.

Problem 4: Low transformation rate

- Try to prepare high-quality plasmid following the tips for the troubleshooting to problem 2.
- Please be aware that the temperature for some centrifuge steps is 4 ˚C, which is key to a successful transformation experiment.
- The PEG solution should be freshly prepared before use, and try to dissolve PEG completely by vortexing.
- Try to place the protoplasts on an ice-water bath immediately after heat-shock without any disturbance.
- The plasmid quality is an influential factor to a successful transformation experiment, and please always use Midi/Maxi-Prep plasmid kit for plasmid preparation.
- The transformed protoplasts should be cultured at 26 ˚C under low light intensity (～11 μmoL/m^2/s).
- The culture time is 16-24 hrs, and culture time longer than 24 hrs will significantly decrease the proportion of cells showing GFP signals.

2.5 Key resources Table

REAGENT or RESOURCE	SOURCE	IDENTIFIER
Bacterial Strains		
Trans1-T1 Phage Chemically Comptent Cell	Transgen Bio Tech	CD501-03
Plant Samples		
C. pumila (WT)	This study	N/A
Aristolochia fimbriata	This study	N/A
Aquilegia ecalcarata	This study	N/A
Physalis floridana	This study	N/A
Petrocosmea sinensis	This study	N/A
Glycine max	This study	N/A
Rehmannia glutinosa	This study	N/A
Lycopersicum esculentum	This study	N/A
Arachis hypogaea	This study	N/A
Plasmids and Recombinant DNA		
pHBT-sGFP(S65T)-NOS	[19]	N/A
p35Smini:GFP	This study	N/A
p4×ABRE-mini:GFP	This study	N/A
p4×mABRE-mini:GFP	This study	N/A
p35S:AREB1	This study	N/A
p35S:GUS	This study	N/A
Chemicals		
KCl	Sigma-Aldrich	V900068
$CaCl_2$	Sigma-Aldrich	C5670
$MgCl_2 \cdot 6H_2O$	Sigma-Aldrich	M2670
NaCl	Sigma-Aldrich	S5886
MES	Sigma-Aldrich	M3671
Mannitol	Solarbio	M8140
BSA	Genview	FA016
PEG4000	Sigma-Aldrich	81240
Cellulase R-10	Yakult	AOV0105
Pectolase Y-23	BioDee	DEC002
Macerozyme R-10	Yakult	AOV0096
Other Commercial Products		
Trypan Blue Stain (0.4%)	Invitrogen	15250061
Fluorescein diacetate (FDA) solution	Coolaber	PPT5351
Rhodamine 123 (rhd 123) solution	Coolaber	SL7132
TIANgel Purification Kit	Tiangen	DP219-02
TIANprep Rapid Mini Plasmid Kit	Tiangen	DP105-02
QIAGEN Plasmid Midi Kit	Qiagen	12143
QIAGEN Plasmid Maxi Kit	Qiagen	12162
In-Fusion HD Cloning Kit	Takara	639649

KOD-Plus-Neo DNA Polymerase	Toyobo	KOD-401
T4 DNA Ligase	Takara	2011A
HindIII-HF	NEB	R3104V
SacI-HF	NEB	R3156V
BamHI-HF	NEB	R3136V
PstI-HF	NEB	R3140V
LB liquid Medium	Coolaber	PM0010L
LB solid medium	Coolaber	PM0020
Murashige & Skoog (MS) Basal Medium	Coolaber	PM1011
Kanamycin	Inalco	1758-9316
Ampicillin sodium	Solarbio	A8180
SV Total RNA Isolation System	Promega	Z3100
RevertAid First Strand cDNA Synthesis Kit	Thermo	K1622
TB Green Premix Ex Taq II (Tli RNaseH Plus)	Takara	RR820A
Others		
0.22 μm sterile filter	Merck Millipore	SLGPR33RB
40 μm nylon cell strainer	Falcon	352340
60 mm ×15 mm tissue culture dish	Corning	430196
50 mL centrifuge Falcon tube	Corning	430828
2.0 mL DNA LoBind Eppendorf Tube	Eppendorf	022431048
Haemocytometer	Easybio	BE6138
Gelzan	Sigma-Aldrich	71010-52-1
Cell counting plate	Easybio	BE6138
24 well cell culture plate	Corning	3524
Glass slide	Sailboat	7105
Glass cover slide	Easybio	10212424C
Vacuum concentrator (minimum to 100 mbar)	ScanVac	ScanSpeed32
Low-temperature centrifugate	eppendorf	5427 R
Orbital shaker	HYCX	CS-100
